# Failure to detect equid herpesvirus types 1 and 4 DNA in placentae and healthy new-born Thoroughbred foals

**DOI:** 10.4102/jsava.v90i0.1736

**Published:** 2019-05-30

**Authors:** Lara J. Brown, Geoff Brown, Julia Kydd, Tom A.E. Stout, Martin L. Schulman

**Affiliations:** 1 Department of Production Animal Studies, University of Pretoria, Pretoria, South Africa; 2 Section of Reproduction, Department of Production Animal Studies, University of Pretoria, Pretoria, South Africa; 3 School of Veterinary Medicine and Science, University of Nottingham, Leicestershire, United Kingdom; 4 Department of Equine Sciences, Utrecht University, Utrecht, Netherlands

**Keywords:** equines, equid herpesvirus type 1 and 4, placentae, foetal membranes, foals, qPCR, latency, Thoroughbred

## Abstract

Equid herpesvirus type 1 is primarily a respiratory tract virus associated with poor athletic performance that can also cause late gestation abortion, neonatal foal death and encephalomyelopathy. Horizontal transmission is well described, whereas evidence of vertical transmission of equid herpesvirus type 1 associated with the birth of a healthy foal has not been demonstrated. This study sampled a population of Thoroughbred mares (*n* = 71), and their healthy neonatal foals and foetal membranes, to test for the presence of both equid herpesvirus types 1 and 4 using a quantitative polymerase chain reaction assay. Foetal membrane swabs and tissue samples were taken immediately post-partum, and venous blood samples and nasal swabs were obtained from both mare and foal 8 h after birth. Neither equid herpesvirus type 1 nor equid herpesvirus type 4 nucleic acid was detected in any sample, and it was concluded that there was no active shedding of equid herpesvirus types 1 and 4 at the time of sampling. Consequently, no evidence of vertical transmission of these viruses could be found on this stud farm during the sampling period.

## Introduction

Herpesviruses typically have a narrow host range and have become highly adapted to their host species (MacLachlan & Dubovi 2011; Schulman 2016). They are enveloped, double-stranded DNA (deoxyribonucleic acid) viruses (Davison 2002, 2010; Griffin, Verweij & Wiertz 2010; MacLachlan & Dubovi 2011) that establish latent infections in their hosts (Griffin et al. 2010), providing a reservoir for continued transmission within the population (MacLachlan & Dubovi 2011).

In horses, multiple herpesviruses have been detected, some of which are associated with clinical diseases. The equid alphaherpesviruses 1 and 4 (EHV-1 and EHV-4) have an economically significant impact on athletic and reproductive performance (Gilkerson et al. 1999). Respiratory disease caused by EHV-1 and EHV-4 is seen most frequently in weanlings and yearlings (Van Maanen 2002) with associated poor performance and loss of training time (Gilkerson et al. 1999). Reproductive losses usually occur because of the late gestation abortions and neonatal foal death caused by EHV-1 (Gilkerson et al. 1999; Van Maanen 2002). Outbreaks of the neurological form of EHV-1 are usually sporadic (Pusterla et al. 2009) and may result in the death or euthanasia of the affected animal (Charlton et al. 1976; Wilsterman et al. 2011).

Primary infection with EHV-1 occurs via the respiratory tract (MacLachlan & Dubovi 2011) following contact with infected secretions from virus-shedding horses (Rusli, Mat & Harun 2014), or contact with an aborted foetus or foetal membranes (Allen et al. 2004). The replication of the virus begins in the epithelium of the upper respiratory tract or conjunctivae and continues in the draining lymph nodes (Allen et al. 2004; Rusli et al. 2014). Within 24 hours (h), EHV-1-infected mononuclear cells are detectable in lymph nodes associated with the respiratory tract (Kydd et al. 1994). Virus-infected cells can be detected in the trigeminal ganglion within 48 h of initial infection (Allen et al. 2004; Slater et al. 1994). Immunologically-naive horses may shed the virus from the nasopharynx for up to 15 days after first exposure, whereas previously exposed horses typically shed for only two to four days (Allen et al. 2004; Burrows & Goodridge 1975). The resultant leukocyte-associated viraemia can then infect the endothelium in the uterus (Allen et al. 2004; Lunn et al. 2009; Rusli et al. 2014). Infection of the endothelial cells of the uterine blood vessels allows for transmission of the virus from the mare to the foetus (Kimura et al. 2004) or placental infarction and detachment (Smith et al. 1992).

The pathogenesis of neurological disease relates to the strong endotheliotropism of virulent strains of EHV-1. Vasculitis and subsequent thrombosis can occur in the central nervous system (CNS), with resultant ischaemic damage and myelomalacia (Friday et al. 2000).

The establishment of latency is a key feature of all herpesvirus infections (Dunowska 2016): EHV-1 becomes latent in the trigeminal ganglia and lymphoid tissue (Slater et al. 1994). A review of literature on latent EHV-1 infection suggested that more than 50% of the horse population is latently infected with EHV-1 (Brown et al. 2007). It has been suggested that shedding of the virus through reactivation of latent infection is an important biological source of the virus (Allen et al. 2004; Edington, Welch & Griffiths 1994). The development of chronic, low-grade infections through reactivation of latency is an effective strategy for EHV-1 to maintain itself within the global horse population (Allen et al. 2004; Brown et al. 2007). Arguably, it is against the interest of the virus to cause the death of its host, and initiating abortion would create a ‘dead end’ in viral replication. An EHV-1 positive abortion or neonatal death may assist horizontal transmission because the abortus or neonate serves as a source of infection. However, a seemingly superior viral evolutionary strategy may be to disseminate the virus via the birth of an infected but viable foal. This may result in immediate infection of vulnerable animals in the same cohort but may also permit the development of latency. Future reactivation events might then continue to disseminate the virus to an even wider population of horses.

In a recent preliminary study, a strong correlation was found between the presence of a major histocompatibility complex (MHC) class 1 B2 allele and pregnancy loss in horses, which was present regardless of the EHV-1 status of the foetus (Kydd et al. 2016). The presence of this allele was found to be a statistically significant risk factor among many risk factors for abortion (Kydd et al. 2016). While this association needs further investigation, it raises the possibility that in mares carrying this particular allele, abortion caused by EHV-1 infection may be an accident, rather than a specific viral propagation strategy. Major histocompatibility complex class I plays a key role in the generation of host immune responses and, *in vitro,* acts as an entry receptor via viral glycoprotein D (Sasaki et al. 2011).

The present study aimed to detect the presence of EHV-1 and -4 DNA in the placentae, blood and nasopharynx of a stud farm’s population of Thoroughbred broodmares and their new-born, viable and healthy foals during a single foaling season.

## Research methods and design

The study population consisted of 71 maiden and multiparous Thoroughbred mares, aged 5–19 years, together with their neonatal foals. All animals were resident on a stud farm near Piketberg, Western Cape, South Africa. The pregnant mares were maintained outside but were stabled during parturition to allow closer supervision.

**FIGURE 1:**
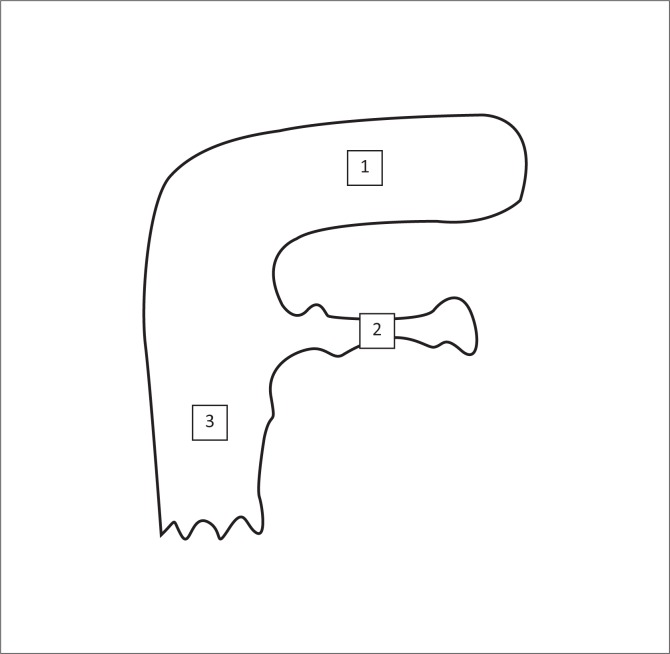
Diagram depicting the three sampling sites on the foetal membranes: (1) pregnant horn, (2) non-pregnant horn, (3) body.

Foetal membrane sampling was performed immediately after placental expulsion. Foetal membranes were inspected to determine their integrity and note any signs of pathology. A dry cotton swab was rubbed over the villous surface of the chorion at three sites, namely pregnant horn, non-pregnant horn and body (Figure 1).

Approximately 8 h after foaling, venous blood samples and nasal swab samples from both mare and foal were collected into EDTA BD Vacutainer® tubes (Becton Dickinson, Johannesburg, South Africa) and 10-cm plastic shafted cotton tipped nasal swabs, respectively.

A duplex quantitative polymerase chain reaction assay (qPCR) was performed for EHV-1 and EHV-4 (Diallo et al. 2006). Nasal and placental swabs were agitated in 0.5 mL of 0.1 M phosphate buffered saline (PBS) (pH 7.4) in a 1.5 mL Pierce™ Microcentrifuge tube (Thermo Fisher Scientific, United States) for 5 seconds (s). Samples were then centrifuged for 60 s at 10 000 G using a desktop centrifuge (Rotanta 460, Germany) to concentrate cellular material and pathogen material, if present. Excess supernatant was removed from each sample container and was discarded to reduce the sample volume. Then, 100 *µ*L of distilled water was added to each container. Samples were then agitated and placed in a temperature-controlled heat block at 95 °C. The 0.1 mL PCR (polymerase chain reaction) plates were prepared in a separate section of the laboratory. The master mix (17 *µ*L per sample) was placed into each sample well of the PCR plate, and a foil seal was placed over the plate. The prepared samples (3 *µ*L) were then added to the individual wells of the plate by introducing the pipette tip through the foil seal. Lastly, the positive and negative controls were added. Nucleic acids extracted from EHV-1 and EHV-4 reference viral cultures obtained from the Equine Virology Research Laboratory, University of Pretoria, were used as positive controls. Endonuclease-free water was used as a negative control. The qPCR was performed according to the manufacturer’s guidelines and followed the standard operating procedure (SOP) of the Veterinary Genetics Laboratory using the Applied Biosystems^TM^ Thermo Fisher Scientific StepOnePlus^TM^ Real-Time PCR System. A cut-off value of 40 cycles (C_t_) was assigned for the detection of viral DNA in the prepared samples.

Ethical approval for the research was obtained from the University of Pretoria’s Animal Ethics Committee (project number V109-16).

## Results

The qPCR failed to detect either EHV-1 or EHV-4 nucleic acid in any nasal swabs collected from the study population of 71 mares and their foals, or from their foetal membranes (Table 1). As EHV-1 and EHV-4 are respiratory tract viruses, the failure to detect viral shedding suggests that cell-associated viraemia in any of the sampled horses was unlikely, and consequently blood samples for serology and viral detection were not tested.

## Discussion

Our study was designed to gather evidence to test the hypothesis that horizontal dissemination is not the only means of transmission of EHV-1 and that vertical transmission is an alternative mechanism for viral propagation. We did not find any evidence of active shedding of EHV-1 or EHV-4 DNA in healthy post-partum mares and their foals nor in the placentae and were, therefore, unable to support this theory. In considering potential pitfalls for our study, an entire batch of false negative samples, as a result of damage to viral DNA during transport, was considered unlikely; prior studies using identical sampling, transport and extraction methods and the same qPCR assay to detect EHV-1 and -4 DNA were successful (Badenhorst et al. 2015; Schulman et al. 2014). Furthermore, the positive control reacted as anticipated. Nevertheless, neither EHV-1 nor -4 viral DNA was detected in this relatively large sample set.

A reported EHV-1 abortion-associated epizootic occurred on the same farm in 2007, with 9 of the then 30 resident pregnant broodmares aborting (Schulman et al. 2012). The current study included five mares that, although present, did not abort during the 2007 outbreak but were probably exposed to infectious EHV-1. An additional mare, present during the previous outbreak, was also resident on the farm but was not sampled because of her barren status in 2016. Given this history, we concluded that at least some mares sampled for the current trial had been previously exposed to EHV-1. Based on this assumption, the mares in the current study may simply not have demonstrated viral recrudescence with subsequent viraemia and shedding (Dunowska 2016). The percentage of latently infected mares was unknown at the time of the study and the farm’s protocol of routine, comprehensive vaccination of pregnant mares may have suppressed viral reactivation and shedding (Goehring et al. 2010; Minke, Audonnet & Fischer 2004).

The detection of active viral shedding in animals that are possibly latently infected presents a challenge that is discussed extensively in the literature. A recent study found a low rate of detection of EHV-1 in adult horses, even among those showing pyrexia and respiratory signs (Pusterla et al. 2016). In another study of 124 hospitalised critically ill horses, no evidence of EHV-1 shedding was detected, although low levels of latency could not be excluded (Carr, Schott & Pusterla 2011). Sonis and Goehring (2013) concluded from a study of hospitalised febrile horses that nasal shedding of EHV-1 and EHV-4 was a rare event, as only one of the 64 febrile horses was PCR positive for EHV-4 and none were positive for EHV-1.

Several studies have reported the time point between birth and weaning at which foals became EHV-1 and -4 positive (Foote et al. 2004; Gilkerson et al. 1999). Foote et al. (2004) showed the presence of EHV-1 and EHV-4 DNA in nasal swabs from a group of foals, some of which were as young as 11 days. The foals were sampled at an average of 40 days old to determine seroprevalence using a glycoprotein G-specific ELISA (27% of the foals). The young age at which these foals seroconverted has two potential explanations: firstly, a very rapid post-partum infection and seroconversion, despite the presence of maternally derived antibody; secondly, as a result of vertical transmission, intrauterine priming may have occurred, leading to rapid seroconversion on exposure immediately after birth. During an EHV-1 abortion storm, EHV-1 was identified by virus isolation in 4 out of 39 foals aged 7–9 days, 3 of which showed no clinical signs (Mumford et al. 1987). In a study by Gardiner and co-workers, EHV-1 was isolated from the chorioallantois of infected mares that gave birth to premature foals, which shed EHV-1 for the first week of life (Gardiner et al. 2012). This repeated discovery of EHV-1 and EHV-4 DNA and infectious virus in very young healthy foals was a significant factor in the justification of the present study.

## Conclusion

A field study sampling a single stud farm with a single management system over one season obviously limits the extrapolation of the findings to either the South African or global horse population. On this particular farm, there was no evidence of active EHV-1 or EHV-4 infection at the time of sampling. Given the cyclic nature of herpesviral disease, repeat sampling in successive breeding seasons or in a breeding season affected by a confirmed EHV-1 outbreak may better represent the actual risk of vertical transmission of EHV-1 in actively shedding horses.

Although this study did not yield any evidence of vertical transmission of EHV-1, the possibility of vertical transmission was not conclusively excluded. Further research is required to address this intriguing hypothesis. Any evidence for vertical EHV transmission would have important consequences for management practices on stud farms and improve our understanding of the dynamics of equid herpesviral disease in horse populations.
